# Robust Spatial Sensing of Mating Pheromone Gradients by Yeast Cells

**DOI:** 10.1371/journal.pone.0003865

**Published:** 2008-12-04

**Authors:** Travis I. Moore, Ching-Shan Chou, Qing Nie, Noo Li Jeon, Tau-Mu Yi

**Affiliations:** 1 Department of Developmental and Cell Biology, University of California Irvine, Irvine, California, United States of America; 2 Center for Complex Biological Systems, University of California Irvine, Irvine, California, United States of America; 3 Department of Mathematics, University of California Irvine, Irvine, California, United States of America; 4 Department of Biomedical Engineering, University of California Irvine, Irvine, California, United States of America; Dartmouth College, United States of America

## Abstract

Projecting or moving up a chemical gradient is a universal behavior of living organisms. We tested the ability of *S. cerevisiae* **a**-cells to sense and respond to spatial gradients of the mating pheromone α-factor produced in a microfluidics chamber; the focus was on *bar1Δ* strains, which do not degrade the pheromone input. The yeast cells exhibited good accuracy with the mating projection typically pointing in the correct direction up the gradient (∼80% under certain conditions), excellent sensitivity to shallow gradients, and broad dynamic range so that gradient-sensing was relatively robust over a 1000-fold range of average α-factor concentrations. Optimal directional sensing occurred at lower concentrations (5 nM) close to the *K_d_* of the receptor and with steeper gradient slopes. Pheromone supersensitive mutations (*sst2Δ* and *ste2^300Δ^*) that disrupt the down-regulation of heterotrimeric G-protein signaling caused defects in both sensing and response. Interestingly, yeast cells employed adaptive mechanisms to increase the robustness of the process including filamentous growth (i.e. directional distal budding) up the gradient at low pheromone concentrations, bending of the projection to be more aligned with the gradient, and forming a more accurate second projection when the first projection was in the wrong direction. Finally, the cells were able to amplify a shallow external gradient signal of α-factor to produce a dramatic polarization of signaling proteins at the front of the cell. Mathematical modeling revealed insights into the mechanism of this amplification and how the supersensitive mutants can disrupt accurate polarization. Together, these data help to specify and elucidate the abilities of yeast cells to sense and respond to spatial gradients of pheromone.

## Introduction

Many obstacles living organisms face involve sensing and responding to changes in the environment. Heterotrimeric G protein systems sense a variety of inputs, from photons in the visual system to chemoattractants in the immune response [1]. These systems have been highly conserved in eukaryotic evolution from fungi to humans [2].

Given their pivotal role in biology and the fact that their receptors (G protein-coupled receptors) are major pharmaceutical targets [3], heterotrimeric G proteins have been the subject of intense investigations. The budding yeast *S. cerevisiae* is a good model organism for studying G protein systems in detail [4] because of the availability of powerful experimental tools (e.g. genetics) and information (e.g. functional genomics). These resources allow for more detailed investigations into the spatial dynamics and regulation. Arguably, the yeast mating pheromone response is one of the best-characterized G protein systems [5], [6].

*S. cerevisiae* undergoes both asexual cell division (budding) and sexual reproduction (mating). The latter occurs when both mating types, *MAT* ***a*** and *MATα*, secrete a pheromone (**a**-factor or α-factor, respectively) to which the opposite mating type expresses the cognate G protein-coupled receptor (GPCR). Exposure to the appropriate mating factor results in cell-cycle arrest and the formation of a mating projection, which is used to reach the potential mate culminating in cellular and nuclear fusion to form a diploid cell [7]. Many of the processes underlying this response such as the signal transduction cascades, cytoskeletal rearrangements [8], cell-cycle arrest, and remodeling of the cell wall, etc., have been described [9].

However to date, the majority of experiments have been performed using α-factor administered in a spatially uniform fashion (i.e. mix cells and pheromone together in a tube). During mating, yeast cells presumably sense spatial gradients produced when the secreted pheromone diffuses toward the partner [10]–[12]. One can argue that previous results characterizing the pheromone response system using isotropic (spatially uniform) α-factor may not reflect true mating conditions. A more realistic input is needed to investigate the spatial dynamics and regulation necessary to sense a gradient, convert this shallow external gradient into a more dramatic intracellular response, and actively correct the direction of the projection extension. However, one must be careful to acknowledge that experimental gradient-generating methods may not be able to reproduce true mating conditions, that such conditions can best be observed in experiments using mating mixtures (e.g. [11]), and that pheromone spatial gradients have not been directly observed during mating, only inferred from what is known about the process.

In 1993, Segall [13] performed a pioneering set of experiments using a micropipette to generate α-factor gradients. He observed that yeast cells could sense the gradient and project toward the source. Alignment accuracy was better at lower concentrations of α-factor (∼10 nM), but significant orientation occurred over a broad range of α-factor concentrations. Pheromone supersensitive mutants displayed decreased accuracy at higher α-factor levels. Segall was able to catalog a wide variety of interesting behaviors in this seminal study. One limitation of these experiments was that quantification of the gradient was indirect and that gradient conditions were not as controllable as ideally desired. A second issue was that most of the data were collected from *BAR1^+^* cells which secrete an α-factor protease thereby influencing the local α-factor concentrations. However, it should be noted that it was not a priority of the author to quantify or characterize the gradient. Here we reproduced and extended the work of Segall and the later work of Vallier, Segall, and Snyder [14].

More recently, Palliwal et al. [15] employed microfluidics chambers to examine gene expression and gradient-sensing during the mating response in yeast. Microfluidics offer the potential advantages of producing stable, reproducible and quantitative gradients. In these experiments, the authors demonstrated the switch-like pheromone activation of gene expression and the important role of the mitogen-activated protein kinase (MAPK) Kss1 to extend the dynamic range of gradient sensing.

In this work, we systematically investigated the abilities of yeast cells for sensing and responding to spatial gradients of α-factor using microfluidics and *bar1Δ* cells. It was possible to observe mating projection at different pheromone concentrations under different gradient conditions. We explored the accuracy, sensitivity, dynamic range, and robustness of gradient-induced polarization. Wild-type cells were good at sensing the spatial gradients, whereas supersensitive mutants showed various defects especially at higher α-factor concentrations. The cells demonstrated several novel strategies for improving the robustness of the response and correcting errors in the direction of the mating projections. In addition, we visualized several proteins involved in pheromone signaling tagged with GFP (green fluorescent protein) to compare polarization in gradients versus spatially uniform α-factor. Finally, we used mathematical modeling to increase our understanding of the data.

## Results

### Generating α-factor gradients using microfluidics and observation of α-factor gradient-induced morphologies

**Figure 1:**
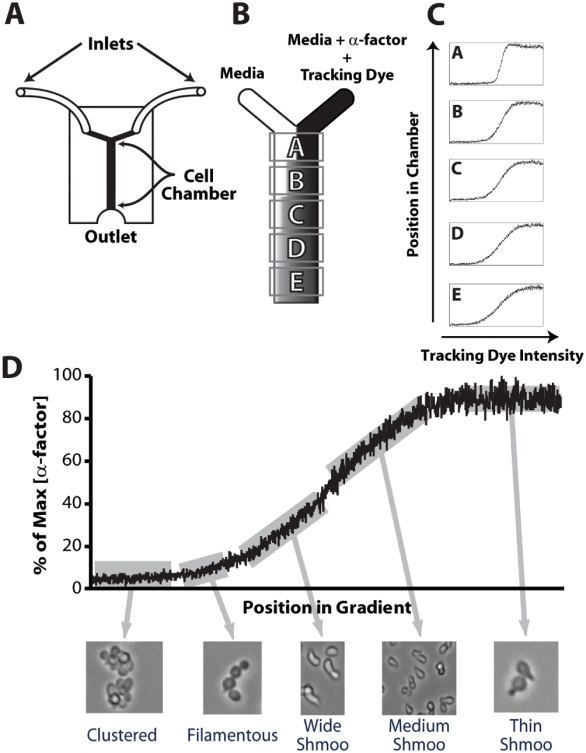
Microfluidics device generates α-factor gradients and response of yeast cells to gradient. (A) Schematic diagram of microfluidics Y-device. There were two inlets, a central channel containing the cells, and an outlet. (B) Media alone at the left inlet and media containing α-factor and Dextran-3000-TRITC (tracking dye) at the right inlet were infused resulting in a gradient across the width of the chamber as the chemicals diffused. Five positions down the length of the central channel, denoted A to E, were visualized. (C) The gradient slope varied depending on the position along the length. The gradient became shallower further down the chamber as the tracking dye and α-factor had more time to diffuse. (D) A variety of cell morphologies were observed depending on the amount of α-factor the cells were exposed to. We observed *bar1Δ* cells in position E in a 0–100 nM gradient.

Microfluidics offer a quantitative and well-controlled method for generating spatial gradients on the micron scale in a reproducible fashion [16], [17]. We used a simple “Y-device”, possessing two inlets converging to a central channel or chamber, to produce an α-factor gradient (Fig. 1A). The device consisted of channels in the polymer poly(dimethylsiloxane) (PDMS) that was attached to a glass slide or coverslip. The cell chamber was 800 µm in width, 15 mm in length, and 100 µm in height. One inlet provided α-factor at a certain concentration and the other provided media without α-factor (Fig. 1B). Laminar flow down the length of the chamber ensured even diffusion across the width of the chamber. The gradient was followed using a 3000 MW fluorescent tracer dye (Dextran-3000-TRITC), which diffused in a similar fashion to α-factor labeled with HiLyte-488 (Supporting Information, Fig. S1). The slope of the gradient varied with the position along the length of the chamber, becoming shallower further down the channel (Fig. 1C).

Exponentially-growing *MAT* ***a*** yeast cells were seeded in the central chamber of the microfluidics device and adhered to the glass bottom using concanavalin A. We used rich media (YPAD) to promote cell growth and to prevent α-factor from sticking to the tubing or chamber. The flow was provided by two syringe pumps at the total rate of 1 µl/min. We heated the chamber to 30° C and exposed the cells to α-factor for 4 hours. With a 10× objective, we could observe across the width of the chamber and took images at five positions along the length of the chamber (positions A through E).

In an initial experiment, we monitored the response of *bar1Δ* cells to a 0–100 nM gradient. The *BAR1* gene encodes for an α-factor protease and strains deleted for this gene do not degrade α-factor. We focused on position E closest to the outlet, which possessed the shallowest gradient. The α-factor concentration gradient went from left (low, 0 nM) to right (high, 100 nM). At the region furthest to the left (lowest α-factor), we saw clustered cells indicative of dividing cells. To the right of the clustered cells, we observed cells that budded distally resulting in filamentous growth [18]. Further to the right, cells were arrested in the cell-cycle and formed a wide mating projection. At the center of the chamber were cells possessing a mid-sized projection. Finally at the right-side of the chamber with the highest α-factor concentrations, we observed cells with a narrow projection. Thus, a wide-range of morphologies and behaviors could be observed in a single experiment (Fig. 1D).

### Yeast mating projections align with α-factor gradient

**Figure 2:**
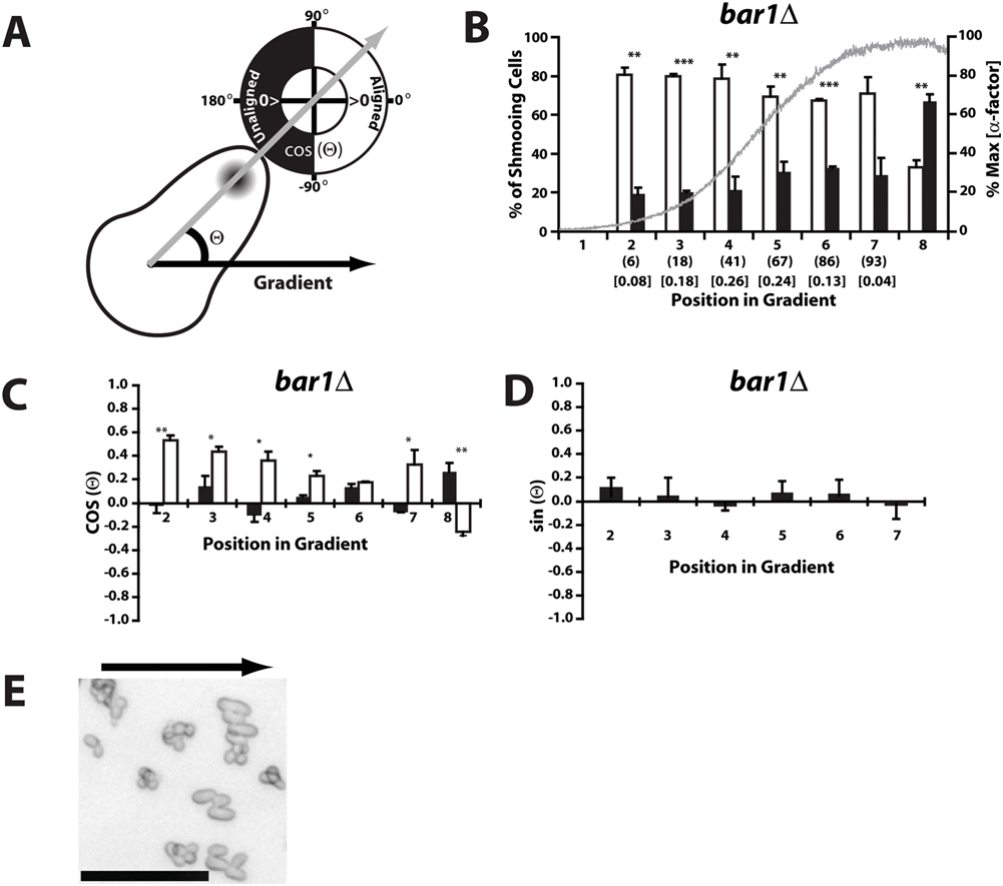
Mating projections align with the gradient. Data is from *bar1Δ* cells at position E in a 0–100 nM gradient after 4 hours in 3 separate experiments (average±SEM). (A) The directional accuracy of projection growth was assessed in two ways: (i) binary discrimination between aligned (−90°<Θ<90°) versus unaligned (90°<Θ<180° or −180°<Θ<−90°) mating projections, and (ii) the projection azimuth which we quantitated as the cosine of the angle Θ between the direction of the gradient and the direction of the projection, as labeled by ConA-Alexa-488. (B) Significant alignment with the gradient. The width of the cell chamber was divided into 8 regions. A significant (* p<0.05, ** p<0.01, *** p<0.001; t-test) percent of projecting *bar1Δ* cells aligned (white bars) with the 0–100 nM gradient when compared to those that did not (black bars). The α-factor gradient, indicated by the fluorescence intensity of the tracer Dextran-3000-TRITC, is represented by the gray line across the bar graph. An estimate of the average concentration of α-factor (nM) in each region is given in parentheses along with the estimated relative slope (nM/µm) in brackets; these data are also in the Materials and Methods. In region 8 the gradient was flat or in the wrong direction caused by edge effects in the chamber, and in region 1 only a few cells did form a mating projection. (C) Comparing the directional response in gradient versus spatially uniform conditions. The projection azimuth Θ of cells exposed to a gradient (white) was significantly (* p<0.05, ** p<0.01; t-test) aligned in the gradient direction, whereas cells exposed to a spatially uniform 100 nM α-factor treatment (black) projected in random directions (cos(Θ)∼0°). (D) Fluid flow did not influence projection direction. Preferential projection growth in the upstream or downstream direction of the microfluidics flow would be indicated by a significantly positive (up against the flow) or negative (down with the flow) values for sin(Θ). We found that sin(Θ) was close to 0. (E) In position A of the microfluidics chamber possessing the steepest gradient, we observed a narrow band of cells (in the middle of the chamber near the boundary between regions 4 and 5) in which most projections were almost perfectly aligned with the gradient. Scale bar = 50 µm. The direction of the gradient was from left (low) to right (high) as shown by the black arrow; cells are *bar1Δ*.

The first question we asked was whether the *MAT* ***a*** yeast cells could sense and respond to the microfluidics α-factor gradient by making a mating projection in the correct direction (Fig. 2A). One measure of accuracy was whether the cells projected correctly toward the right (aligned up the gradient) or incorrectly to the left (unaligned down the gradient). Initially, we examined the *bar1Δ* cells in position E in the 0–100 nM gradient after 4 hours. The width of the chamber was divided into 8 regions, numbered from left to right. In region 1, the level of α-factor was too low to induce projection formation, and in region 8, the gradient was flat or in the wrong direction; in addition, there were edge effects from the PDMS borders in these two regions. In regions 2 to 7, the cells significantly projected in the correct direction in three separate trials (Fig. 2B), thus indicating that the cells can correctly sense the gradient.

In these experiments, it was important to select single cells that were not in clusters. We noticed that cells (*bar1Δ* and *BAR1^+^*) in clusters tended to project away from the center of the cluster regardless of the gradient (data not shown). This significant tendency could obscure the ability to sense the α-factor gradient.

A more informative measure of directional sensing was the angle Θ of the mating projection with respect to the gradient (Fig. 2A). We calculated cos(Θ), which was positive if the projection was in the correct direction, and 1 if perfectly aligned. More precise determination of the mating projection direction was aided by using the fluorescent dye ConA-Alexa-488 to label the growing projection; this dye did not perturb the directional sensing (Supporting Information, Fig. S2). In the experiment described above, the cos(Θ) was positive indicating alignment with the gradient, but the average value was less than 0.6 (arccos(0.6) = 53°) indicating that the directional sensing was far from perfect. An important control experiment was measuring alignment when adding 100 nM α-factor in a spatially uniform manner to the microfluidics chamber; in this isotropic case, we observed almost random orientation of the yeast mating projections (Fig. 2C). Note that yeast cells will project under spatially uniform pheromone conditions because of the presence of an internal cue (bud scar) that directs polarization independent of the external gradient [10], [19]. Finally, we examined whether projection direction (Θ = angle of projection with respect to gradient direction, left to right) was influenced by the fluid flow in the chamber (top to bottom). We measured whether the projection was preferentially up (sin(Θ)>0, against the flow) or down (sin(Θ)<0, against the flow) the chamber. We found that sin(Θ) was close to 0 indicating an absence of a significant preference in projection orientation with respect to the flow direction (Fig. 2D).

Interestingly, in the chamber we observed one place with high accuracy where most cells were almost perfectly aligned with the gradient (cos(Θ)∼1, Fig. 2E). This was only observed in Position A where the gradient was quite steep. This result suggests that steep gradient slopes promote the most accurate sensing and response.

### Wild-type and *bar1Δ* cells exhibit sensitivity and broad dynamic range for gradient-sensing

**Figure 3:**
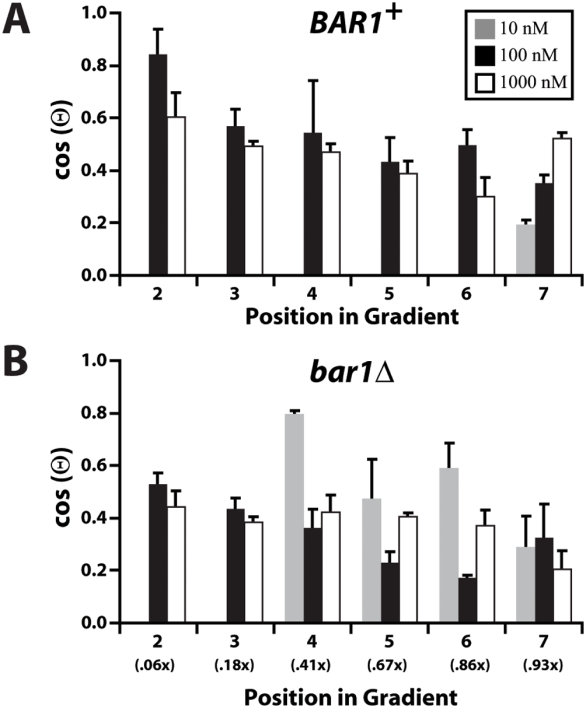
Assessing the gradient sensitivity and dynamic range of the spatial sensing response. The projection azimuths of (A) wild type *BAR1^+^* and (B) *bar1Δ* cells were determined in three different α-factor gradients: 0–10 nM (gray), 0–100 nM (black), 0–1000 nM (white). Most *BAR1^+^* cells did not project at α-factor levels below ∼10 nM, but showed good directional accuracy even in region 7 of the 0–1000 nM gradient where the average pheromone concentration was ∼900 nM. The *bar1Δ* cells showed reduced overall accuracy compared to *BAR1^+^* cells in the 0–100 nM and 0–1000 nM gradients, but the directional accuracy was still significant at the higher concentrations. The concentration of α-factor in each region is shown in parentheses where *x* is the maximum concentration for that gradient (i.e. *x* = 10, 100 or 1000 nM). Both types of cells could sense the gradient direction where the relative slope was shallowest in regions 6 and 7 of all three gradients. Three independent experiments were performed (average±SEM).

We tested the directional projection formation of *BAR1^+^* (Fig. 3A) and *bar1Δ* (Fig. 3B) cells over three different gradient ranges in which the upper concentration was varied: 0–10 nM, 0–100 nM and 0–1000 nM. First for the *bar1Δ* strain, as described above, we examined cells in Position E of the chamber, divided the width into 8 regions, and measured the angle of the projection. Projection accuracy was highest at the lowest concentration gradient (0–10 nM). In this range, the α-factor levels were closest to the dissociation constant of α-factor receptor (K_d_∼5 nM), and represent the levels at which the receptor can best sense differences in ligand concentration. Below 2 nM, many of the cells were still dividing and did not make a projection. At approximately 5 nM, we observed the most accurate projection in which cos(Θ)∼0.8.

For the 0 to 100 nM gradient, we observed the highest accuracy at the lower concentrations and there was a trend in which the accuracy declined as we moved right across the chamber to sections at higher average concentrations and shallower relative slopes. Interestingly, we observed gradient detection in the 0 to 1000 nM gradient even at the high end (close to 1 µM) where we expect receptors at both the front and back of the cell to be almost completely bound with ligand. Thus, the *bar1Δ* cells showed the ability to make projections that sense gradients roughly from 4 nM to 1000 nM, which is more than two orders of magnitude dynamic range of spatial sensing (Fig. 3B).

We repeated the above experiments over the three gradient ranges in *BAR1^+^* cells (Fig. 3A); Bar1 is a protease in **a**-cells that degrades α-factor. For the 0–100 nM and the 0–1000 nM gradients, the *BAR1^+^* cells displayed better accuracy than the *bar1Δ* cells, thus highlighting the role of Bar1 to improve gradient-sensing at higher ligand concentrations [13]. Bar1 acts even in the constant flow of the microfluidics chamber, which presumably washed free Bar1 protease away from the cells. This finding is consistent with the observation that some fraction of the Bar1 protease may be attached to the cell wall [20]. Finally, for both sets of cells, the directional accuracy was best when the overall concentration of α-factor was closest to the receptor *K_d_*, indicating the average level of α-factor exposure was an important determinant of accurate gradient-sensing.

How shallow of a gradient can the yeast cell sense? We represented the slope as relative to the average concentration (*L_mid_*) at that position (*z*): *L_slope_*__*rel*_ = *L_slope_*/*L_mid_* = *d* ln *L*/*dz*. In the middle of the chamber we estimated the slope to be 0.5% µm^−1^, and in regions 6 and 7, the slope was approximately 0.1% µm^−1^, which was a 0.5% difference in ligand concentration between front and back for a 5 µm long cell. An important technical point about the experiments was that α-factor was not being lost sticking to the sides of the tubing or chamber so that the estimates of the α-factor concentration were accurate (Supporting Information, Fig. S3).

### Supersensitive mutants show decreased accuracy at higher concentrations of α-factor

G-protein activation and G-protein deactivation are two critical control points in the dynamics of G-protein signaling. We examined gradient-sensing and projection formation in mutants in which regulation of these processes was altered. The absence of the RGS protein Sst2 (*sst2Δ*) reduces G-protein deactivation; deletion of the C-terminal tail of Ste2p (*ste2^300Δ^*) reduces receptor down-regulation. Both mutants are supersensitive in their response to α-factor with the *ste2^300Δ^* mutant approximately 10-fold more sensitive and the *sst2Δ* mutant approximately 100-times more sensitive to pheromone by the halo assay. It is important to distinguish the sensitivity to the gradient slope (i.e. ability to detect shallow gradients) discussed in the last section from the sensitivity of the pheromone response to the absolute levels of α-factor. We examined both mutations in a *bar1Δ* background, whereas previous work [13], [14] examined the supersensitive mutations primarily in a *BAR1^+^* background.

**Figure 4:**
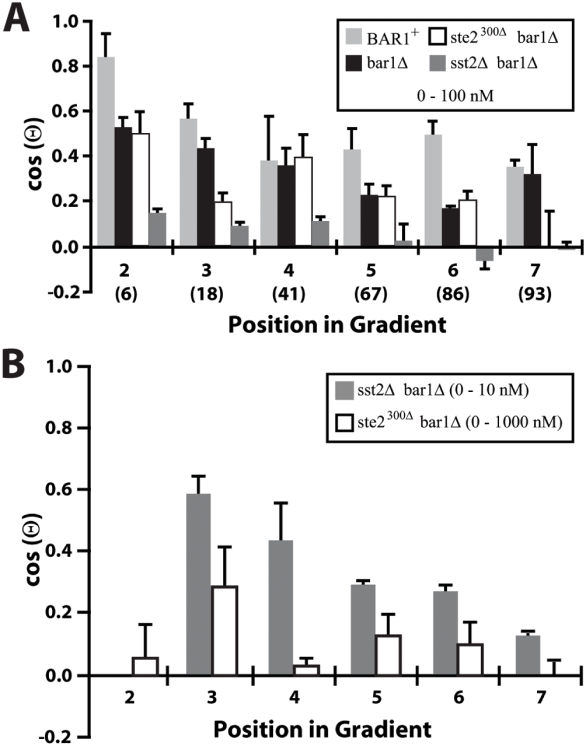
Gradient-sensing of supersensitive *sst2Δ* and *ste2^300Δ^* strains in *bar1Δ* background. (A) In a 0–100 nM gradient, we measured the directional accuracy, cos(Θ), of *BAR1^+^* (light gray), *bar1Δ* (black), *ste2^300Δ^ bar1Δ* (white), and *sst2Δ bar1Δ* (dark gray) cells. The *BAR1^+^* and *bar1Δ* data are reproduced from Fig. 3. The *sst2Δ bar1Δ* cells exhibited a severe gradient-sensing defect, whereas the *ste2^300Δ^ bar1Δ* cells resembled the *bar1Δ* cells. An estimate of the average concentration of α-factor (nM) in each region is given in parentheses. (B) Response of *sst2Δ bar1Δ* cells in a 0–10 nM gradient (dark gray) and *ste2^300Δ^ bar1Δ* cells in a 0–1000 nM gradient (white). The *sst2Δ* strain showed good directional sensing in this lower range of α-factor concentrations. The sensing of the *ste2^300Δ^* cells was poor at the higher pheromone levels. Three independent experiments were performed (average±SEM).

The gradient-sensing and response defect in the *sst2Δ bar1Δ* strain was quite dramatic. In the 0–100 nM gradient, most cells oriented randomly with respect to the gradient direction (Fig. 4A). However at reduced α-factor concentrations, in the 0–10 nM gradient, there was directional sensing (Fig. 4B). These data were consistent with the results of Segall [13]. The defect was less severe in the receptor mutant. The *ste2^300Δ^ bar1Δ* cells showed very similar projection accuracy to the *STE2^+^ bar1Δ* cells except in region 7 of the 0–100 nM gradient (Fig. 4A). The defect was more evident in the 0–1000 nM gradient in which accuracy was slightly above random, but not comparable to the *STE2^+^* cells (Fig. 4B). These data were consistent with the results of Vallier et al. [14] which showed mild but significant gradient-sensing defects in *ste2-T326* cells and more severe defects in *ste2Δ296* cells. Overall, there was a correlation between the degree of supersensitivity of the two mutants and the ability to sense gradient direction at higher α-factor concentrations. The *sst2Δ bar1Δ* cells (100-fold supersensitive) showed gradient sensing up to the 0–10 nM gradient and the *ste2^300Δ^ bar1Δ* cells (10-fold supersensitive) showed directional sensing up to the 0–100 nM gradient, whereas *bar1Δ* cells could perform spatial sensing up to the 0–1000 nM gradient.

The *sst2Δ* and *ste2^300Δ^ bar1Δ* cells exhibited additional defects in the formation of mating projections in response to a gradient. First, the mating projections were misshapen; they were irregular, broader, and bent at high concentrations compared to wild-type projections, which were more regular, narrow and straight at the comparable concentrations. Despite the misshapen projections, we were able to determine the projection direction for these mutants (Supporting Information, Fig. S4). Vallier at al. [14] originally observed abnormal projection morphologies in receptor C-terminal mutants. Second, in the mutant strains even at position A, there was no evidence of the highly aligned phenotype (Fig. 2D); thus, they were unable to exhibit the high accuracy projection behavior at steep gradient slopes of wild-type cells. Third, neither mutant strain was able to form multiple projections at high concentrations of α-factor. Taken together these data argue that Sst2 and receptor modification via the C-terminal tail may have additional roles in gradient-sensing beyond preventing supersensitivity to α-factor.

### Error correction and robustness strategies

**Figure 5:**
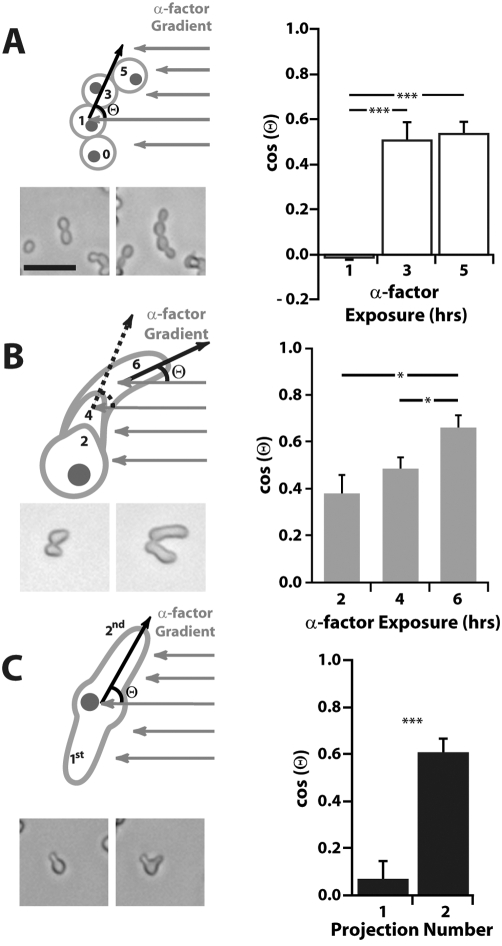
Pheromone gradient-sensing robustness strategies: schematic diagrams, data, and sample images. (A) At α-factor concentrations between 1 and 4 nM, many cells (*bar1Δ*) began to bud distally in a filamentous fashion. These filaments sensed the gradient as indicated by the direction of the bud relative to the mother cell and the resulting orientation was significantly (*** p<0.001; t-test) toward higher concentrations of mating factor. Scale bar = 50 µm. (B) At α-factor concentrations between 5 nM and 40 nM, cells made a wide mating projection that bent in the direction of the gradient. Determination of projection direction was aided by use of ConA-Alexa-488, which labeled the growing projection. Cells were imaged after 2, 4, and 6 hours of exposure. At each succeeding time interval the alignment with the gradient, cos(Θ), increased (* p<0.05; t-test). (C) At α-factor concentrations above 50 nM, if the initial projection was unaligned with the gradient, then instead of altering the projection to orient it toward the gradient, the cell abandoned its first attempt and formed a second projection after 6 hours, which was significantly (*** p<0.001; t-test) more aligned. Three independent experiments were performed (average±SEM).

There were limitations to the response of cells to an α-factor gradient in terms of the directional accuracy of the initial projection, and whether a mating projection was created at all. At low concentrations of α-factor, most cells did not form a mating projection, and instead continued to divide. However, at approximately 2 nM, many cells did not bud axially to create cell clusters, but instead formed filaments that resulted from distal budding away from the birth site. Erdman and Snyder originally characterized this behavior [18]. We asked whether this pheromone-induced filamentous growth sensed and responded to the gradient. After one hour of α-factor exposure, the direction of new buds was random with respect to the gradient. Presumably, many of these buds were committed before the pheromone gradient was applied. After 3 hours, new buds did align with the gradient (cos(Θ)∼0.5) in a significant fashion. The same directionality was observed after 5 hours of gradient treatment when the next bud was made (Fig. 5A). Thus, pheromone-induced filamentous growth does sense and respond to the gradient.

In the range of 5 to 40 nM of α-factor in a 0–100 nM gradient, many cells formed broad projections that bent to follow the gradient. This adaptive response originally described by Segall [13] is illustrated in Fig. 5B. We observed cells at two-hour intervals (2, 4, and 6 hours) and at each succeeding time point, the directional accuracy improved as the projection bent in the correct direction.

Above 50 nM of α-factor, many cells made second projections. We tested whether some second projections represented a probabilistic “correction” of a less accurate first projection. From a sample of cells containing two projections after 6 hours, we found that the first projection was almost randomly oriented, whereas the second projection showed good alignment with the gradient (cos(Θ)∼0.6, Fig. 5C). Thus, there is likely to be an active sensing mechanism involved in the formation of multiple projections, rather than the projections emerging in random directions.

### Polarization of proteins in gradients versus spatially uniform α-factor

We compared the polarization of four different proteins tagged with GFP – Ste2-GFP, Ste18-GFP (Gγ), and Ste20-GFP (kinase activated by Cdc42), Spa2-GFP – under gradient and non-gradient conditions. Both sets of experiments were performed in the microfluidics chamber, and the cells were imaged after 2 to 4 hours of exposure. For the isotropic conditions we used 50 nM of alpha-factor applied to both inlets, and for the gradient conditions we observed cells at position E in the middle regions of the chamber of a 0–100 nM gradient, where the average α-factor concentration was approximately 50 nM.

**Figure 6:**
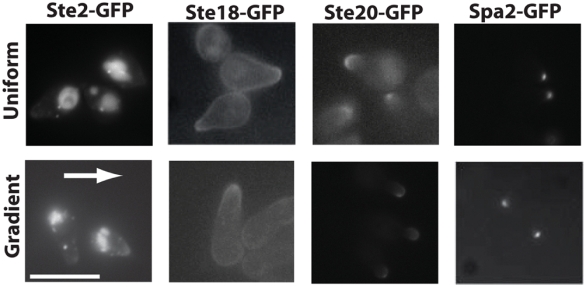
Fluorescence images of Ste2-GFP, Ste18-GFP (Gγ), Ste20-GFP and Spa2-GFP under non-gradient (uniform, 50 nM) and gradient (0–100 nM) α-factor conditions (*bar1Δ*). The polarization patterns were qualitatively similar under the two treatments. Cells were exposed to α-factor in a microfluidics chamber for 2 to 4 hours until pronounced mating projections were observed. Gradient-responding cells were chosen toward the middle of the gradient (∼50 nM) at position E. The gradient direction was from left (low) to right (high) indicated by the white arrow. The large bright structure at the back of the cell in the Ste2 images was the vacuole containing internalized Ste2. Spa2 was tightly localized at the projection tip (the cell body cannot be seen). Spa2 and Ste20 were more polarized than Ste2 and Ste18. Scale bar = 10 µm.

The point of this experiment was to examine the formal possibility that polarization in an external gradient would be significantly and dramatically different from polarization under uniform conditions which would be directed by an internal cue, the bud scar. Qualitatively, we did not observe any significant differences in the polarization of the proteins in the gradient versus the spatially uniform alpha-factor (Fig. 6). These results support the common assumption that the same basic machinery and events are taking place in yeast cells exposed to α-factor whether or not in a gradient [5], [6]. These data, however, do not exclude the possibility of more subtle gradient-specific behaviors under a subset of gradient conditions.

A second goal of this experiment was to examine the extent of polarization induced by the gradient to address the issue of amplification required for tight localization of protein components, and this question is explored in the modeling below. Interestingly, we observed different degrees of polarization for the various proteins (whether in a gradient or not). Spa2 [21] was the most polarized forming a tight spot (polarisome [22], [23]), and Ste20 [24], [25] also displayed a highly-localized appearance. Alpha-factor receptor [19], [26] was significantly polarized at the front, but in many cells, there were some receptors in the cell body, and much of the internalized Ste2-GFP could be found in the vacuole, which was located at the back of the cell. Finally, Ste18 [19], [27] displayed a similar extent of polarization as Ste2 with distinct localization in the mating projection, but still substantial staining in the membrane of the cell body. Thus, for at least 2 proteins (Spa2 and Ste20) there was dramatic polarization whose directionality was influenced by the gradient. This localization is indicative of substantial amplification in which a shallow external gradient is amplified to produce a steep internal gradient of certain proteins.

### Computer simulations to determine sources of amplification for polarization

We used computer simulations to interpret some of the data described above. Based on previous work [28], we constructed a simplified model representing the spatial dynamics of the heterotrimeric and Cdc42 G protein cycles. The first four equations represent the dynamics of the heterotrimeric G protein cycle, and the last two equations represent the dynamics of the Cdc42 G protein cycle. The input is the ligand α-factor (L) and the output is active Cdc42 (C42a).

In the heterotrimeric G-protein cycle, the ligand α-factor (L) binds α-factor receptor (R) to form the active receptor complex (RL). The RL species catalyzes the activation of the heterotrimeric G-protein (G) to form active α-subunit (Ga) and free Gβγ (Gbg). Ga is deactivated to form inactive α-subunit (Gd), which binds to Gβγ to reform the heterotrimer. In the Cdc42 cycle, the level of Gβγ affects the rate of activation of Cdc42 (C42) to its active form (C42a) through a cooperative Hill term (*k_0_* term). In addition, there is a positive feedback term in which C42a stimulates it own activation (*k_1_* term). This expression describes the positive feedback loop involving Cdc42, Cdc24, and the scaffold protein Bem1 [29]. The negative feedback loop is implemented through the action of the Cdc42-activated kinase Cla4 which is known to phosphorylate and down-regulate the Cdc42 activator Cdc24 [30].
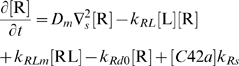
(1)

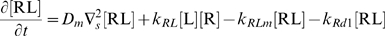
(2)

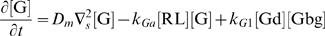
(3)

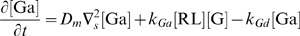
(4)

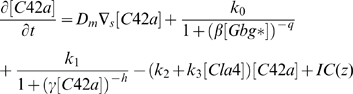
(5)

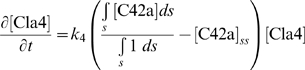
(6)


The full model is described in Appendix S1 (Supporting Information). Polarization was influenced by both an internal cue (*IC*(*z*)) in Eq. (5), describing signaling arising from the bud scar through the G protein Bud1 [10], [19], and the external α-factor gradient. The cell was described as an ellipsoid that is represented along its major axis in one-dimension.

We focused on better understanding the amplification and dynamic range in wild-type cells, as well as the mutant phenotypes. The external gradient of α-factor resulted in an internal gradient of active G-proteins (Gα-GTP and Gβγ). This internal gradient was then amplified by the Cdc42 dynamics described in equation (5). In particular, the equation contains a cooperativity term arising from assembly of higher-order protein complexes (e.g. polarisome) and represented by the Hill term 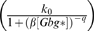, and a positive feedback term 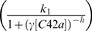 representing the dynamics from the positive feedback loop involving Cdc24, Cdc42, and Bem1 among others [29]. One question is whether both terms are necessary for polarization.

**Figure 7:**
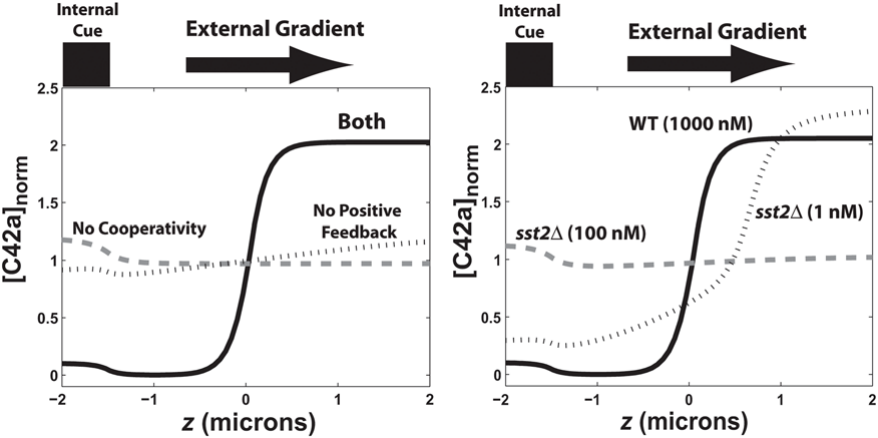
Computer simulations of gradient-induced cell polarization. The normalized concentration of active Cdc42, [C42a]_norm_, was plotted against the axial length of the cell. An internal cue signal was at the left, and the gradient of α-factor pointed from left (low) to right (high). The relative slope of the gradients (*d* ln *L*/*dz*) was 1% µm^−1^. (A) Polarization in response to a gradient (*L_mid_* = 10 nM, *L_slope_* = 0.1 nM/µm) with (i) both cooperativity and positive feedback (*k_0_* = *k_1_* = 0.1 s^−1^; *q* = 200, *h* = 8; solid line), with (ii) only positive feedback and no cooperativity (*q* = 1; gray dashed line), and with (iii) only cooperativity and no positive feedback (*h* = 0, no polarized receptor synthesis; dotted line). (B) Modeling the dynamic range of polarization in wild-type and mutant cells. Simulated wild-type (*bar1Δ*) cells polarized in gradients at high concentrations of α-factor (*L_mid_* = 1000 nM; solid line). The *sst2Δ* (*bar1Δ*) cells in simulations did not polarize at 100 nM (gray dashed line), but did polarize at lower concentrations, 1 nM (dotted line).

In this model, we needed both cooperativity and positive feedback to achieve gradient-induced polarization observed in the experiments (Fig. 7A); the normalized levels of active Cdc42 ([C42a]_norm_) were plotted along the length of the cell. A pure cooperativity model (h = 0, no receptor polarization) could not sufficiently amplify the external gradient (Fig 7A). Without cooperativity (q = 1), there was no sensing of the gradient, and instead there was a minor response to the internal cue, which was to the left.

This balance of cooperativity and positive feedback was able to reproduce both the broad dynamic range of wild-type cells and the reduced dynamic range of the mutant cells. In the balanced model in which *k*_0_ = *k*_1_ = 0.1 s^−1^ (*q = 200*, *h = 8*), the cells could sense the gradient and polarize at concentrations as high as 1 µm α-factor as was observed in the experiments. On the other hand, simulations of *sst2Δ* (*bar1Δ*) cells showed defective gradient-induced polarization at 100 nM average α-factor concentration (Fig. 7B). The *sst2Δ* mutation caused a saturation of active G-proteins so that the internal gradient was too shallow (G-proteins were almost fully active at both front and back) to be amplified by the downstream mechanisms. The relative slope of active G protein was 2.5×10^−4^ µm^−1^ in *bar1Δ* cells, but 5×10^−6^ µm^−1^ in *sst2Δ bar1Δ* cells in the simulations with *L_mid_* = 100 nM. At lower concentrations of α-factor (1 nM) in *sst2Δ* cells, the level of active G-proteins was not saturated, and the internal gradient was sufficiently steep (2×10^−3^ µm^−1^) to allow polarization (Fig. 7B). Simulations showed a similar polarization defect in *ste2^300Δ^* cells at 100 nM α-factor, but not at 10 nM. These modeling data argue that the amplification necessary for polarization in yeast arises from a combination of cooperativity and positive feedback, and that regulation is important for preventing excess G-protein activation, especially in gradients in which the average α-factor concentration is high. Furthermore, the model predicts a high degree of cooperativity (*q*>100) in this signaling system, which may arise from the cooperative assembly of a large complex or from a cascade of cooperative binding reactions. Indeed, there are several scaffold proteins (e.g. Ste4, Ste5, Bem1) which bind multiple different proteins that could form a network of interactions, and in addition, there are also large structures such as the polarisome [9], [21] composed of many subunits that could give rise to highly cooperative behavior.

## Discussion

In this work, we used microfluidics to generate mating pheromone gradients and tested the ability of yeast cells to sense and respond to the gradients by making a mating projection. The microfluidics and *bar1Δ* strains enabled better quantification of input conditions compared to previous studies. We found that yeast cells were very good at sensing spatial gradients capable of high accuracy when the concentration was close to the *K_d_* of alpha-factor receptor (∼5 nM) and when the gradient slope was steep. Although accuracy decreased at higher α-factor concentrations and shallower slopes, the cells were still able to sense the gradient-direction over a broad range of concentrations and gradient slopes, even at concentrations as high as 1 µM and relative slopes (*d* ln *L*/*dz*) as shallow as 0.1% µm^−1^.

Assuming 10,000 receptors/cell [31] and that the receptors have achieved steady-state binding with a fixed *K_d_* of 5 nM, then in the middle of the 0–1000 nM gradient the average concentration was 500 nM and the absolute slope was 2.5 nM µm^−1^. These numbers translate to 4951 occupied receptors in the front-half of the cell and 4950 occupied receptors in the back-half of the cell. Thus, given the above assumptions which need experimental validation, yeast cells exhibit the ability to discriminate, albeit imperfectly, the spatial difference of a single occupied receptor or less.

The overall robustness of projection formation was improved by specific adaptive mechanisms that depended on the α-factor concentration. At very low concentrations when no mating projection formed, cells underwent filamentous growth up the gradient. One can speculate that eventually a daughter cell would be close enough to the source to initiate a mating projection, resulting in long-distance mating. At low concentrations the projection was wider and capable of bending in the direction of the gradient. At high pheromone concentrations, the cell had a greater tendency to make a second projection if the first projection was in the wrong direction. It should be noted that the relationship between these “robust” behaviors and mating efficiency has not been clearly demonstrated.

Mutations that impaired the down-regulation of heterotrimeric G protein activation exhibited gradient-sensing defects that correlated with the supersensitivity of the mutants cells to α-factor. We used computer modeling to describe a scenario in which saturation of the system minimizes the spatial differences in active G-proteins, resulting in an internal active G-protein gradient that is too shallow to be amplified by a downstream mechanism. The generic model exhibited the dramatic amplification and broad dynamic range of gradient-sensing in wild-type cells, as well as defective polarization for “mutant” parameter values. Further research is needed to elaborate this simple model with more realistic mechanistic terms and data, but it provides a starting point for future comparisons. For example, one area of improvement is adding more mechanisms relating to the down-regulation of Ste2. These include receptor modification and endocytosis [32], [33], the role of auxiliary proteins such as Afr1 [34], [35], possible pre-coupling of G-proteins to receptor [36], and the interaction between Sst2 and Ste2 [37]. Indeed, more accurate modeling of the changes in receptor numbers and location is a top priority for future research because of the significant potential impact of these spatial dynamics on gradient-sensing and response.

An interesting question is whether the fundamental behavior of the yeast cells is different in gradients versus spatially uniform α-factor. Examining the polarization of four proteins tagged with GFP suggests no. Furthermore, the dramatic polarization of Ste20 and Spa2 argues for the presence of mechanisms capable of producing substantial amplification of the external spatial gradient. One can rationalize that the heteroterimeric G-protein cycle components Ste2 and Ste18 are less polarized so that they can better sense changes in the direction of the gradient and make a second projection if necessary.

The cells in the microfluidics chambers were under constant flow. We tested flow rates both lower (0.5) and higher (5) than 1 µl/min with no apparent change in the cell behavior; however, at lower flow rates it was harder to maintain a stable gradient. In addition, we did not observe any bias in the projection direction with respect to the flow direction. Qualitatively, the transcriptional (*P_FUS1_-GFP*) and morphological responses of cells exposed to spatially uniform α-factor in a microfluidics chamber were similar to cells responding to α-factor in an incubation chamber without flow (data not shown). Finally, many of our results were consistent with results from other labs using different microfluidics [15] or micropipette [13], [14] techniques to generate gradients.

On the one hand, some of the impressive features of yeast gradient-sensing help to explain the high efficiency of mating. For example, the expression of the α-factor protease Bar1 improves performance at high pheromone concentrations. Presumably, Bar1 exerts its beneficial effect by reducing the concentration of α-factor in the vicinity of the cell to levels closer to the K_d_ of the receptor, resulting in more optimal spatial sensing. On the other hand, the imperfect nature of the observed gradient-sensing also argues for additional strategies to ensure robust mating, because a misalignment of mating projections could prevent cell fusion. The sensing of α-factor represents only part of the whole mating process. One must also consider the directional secretion of α-factor by α-cells, as well as the directional sensing and secretion of **a**-factor. Ultimately, it is important to connect the gradient-sensing performance monitored by microfluidics with the mating performance measured by mating assays.

In the future, we plan to address additional issues of robustness relating to spatial sensing of gradients. What is the effect of internal perturbations such as mutations in other genes of the mating response system? Also, how do external perturbations such as noise in the gradient affect sensing? In addition, what are the limits to the gradient sensitivity and dynamic range? Finally, how well do yeast cells track a moving signal source? Finally, from a modeling perspective, additional spatial dynamics need to be included such as receptor down-regulation, spatial dynamics of Sst2, regulation of the G-proteins, and the role of auxiliary regulators such as Afr1.

Heterotrimeric G protein systems not only monitor the absolute levels of input signals, but also detect spatial and temporal changes in these signals [38]. For example, retinal rod cells can detect spatial contrast in an image over 2 log units of background light intensity, and cone cells over 5 to 6 log units [39]. As comparison, we have shown that yeast cells could sense spatial chemical gradients over 3 log units of pheromone concentration from 1 nM (filamentous growth) to 1000 nM with slopes as shallow as 0.1% µm^−1^, and produced dramatic polarization of proteins in the correct direction. These performance benchmarks can shed light on the logic of the complex design of the yeast mating response and other heterotrimeric G protein systems.

## Materials and Methods

### Plasmids and strains

All yeast strains were isogenic derivatives of W303 or S288C (BY4741). Genetic techniques were performed according to standard methods [40]. Details on strains are presented in Table S1 (Supporting Information).

### Microfluidics devices

Microfluidic devices were fabricated in poly(dimethylsiloxane) (PDMS) using soft lithography [17]. The PDMS was bonded to a glass slide or cover slip. The device was in a Y-shaped configuration, and the central chamber was 800 µm in width, 15 mm in length, and 100 µm in height. Microfluidic devices were treated with concanavalin A (Con A, Sigma) to help the yeast cells adhere to the glass.

### Microfluidics experiment

Yeast cells were grown in YPAD media (yeast extract-peptone-dextrose (YPD) media supplemented with adenine). We added 25 µg/ml of ConA-Alexa488 (Molecular Probes) to the YPAD to mark the growing projection. For one of the two inlets, the 2 mL of media was supplemented with 500 nM Dextran-3000-TRITC (Molecular Probes) and α-factor (10 nM, 100 nM or 1000 nM).

The microfluidics device was placed on an inverted Nikon Eclipse TE300 microscope and imaged with a 10× objective for cell morphology, and 100× objective for fluorescence images of GFP-tagged proteins. The device, stage, microscope, and media were all heated to 30 degrees.

Two Versa Pump 6 syringe pumps (Kloehn, Las Vegas, NV, USA) were connected to the microfluidics device using PE-20 tubing (Becton Dickinson). Each pump was run at a rate of 0.5 µl/min, and were controlled by the LabVIEW program (National Instruments). Gradients were set up over a five-minute period prior to data collection.

The average gradient properties at position E of the microfluidics chamber were as follows for regions 2 to 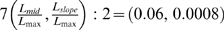, 3 = (0.18, 0.0018), 4 = (0.41, 0.0026), 5 = (0.67, 0.0024), 6 = (0.86,0.0013), and 7 = (0.93, 0.0004), *L*_max_ = maximum α-factor concentration. Each region was 100 µm in width.

### Imaging and image analysis

Images were acquired at 15 minute intervals using a CCD camera (Hamamatsu ORCA-2) connected to the Nikon inverted microscope controlled by the Metamorph software (Molecular Devices) and containing an automated stage. Five positions were imaged (0, 2.5, 5, 7.5 and 10 mm relative to the top of the central chamber) at three wavelengths (bright-field, FITC, TRITC) over four to six hours. The gradient profile at each position in the cell chamber was determined using the tracking dye Dextran-3000-TRITC. The dye ConA-Alexa488 was used to determine the direction of the mating projection. Image analysis was manually performed within ImageJ [41] and CellProfiler [42].

## Supporting Information

Figure S1 Gradient profile of alpha-factor labeled with Hylite-488 compared to Dextran-3000-TRITC. In separate experiments, we monitored the fluorescence profile in the microfluidics chamber of (a) alpha-factor-Hylite-488 (blue) and (b) Dextran-3000-TRITC. We show data from positions A and E in the chamber. The agreement was good indicating that both dyes possess similar diffusion properties in the chamber.
(0.70 MB EPS)

Figure S2 Mating projection directional accuracy in the presence or absence ConA-Alexa-488. The directional accuracy of mating projection growth was assessed by binary discrimination between aligned versus unaligned projections; percent aligned are shown on the y-axis. Results in the presence of ConA-Alexa-488 are the black bars (reproduced from Figure 2B), and the results in the absence of ConA-Alexa-488 are the white bars. Data is from *bar1Δ* cells at position E in a 0–100 nM gradient after 4 hours in 3 separate experiments (average±SEM).
(0.57 MB EPS)

Figure S3 Dose-response of *P_FUS1_-GFP* strain in the microfluidics chamber. Cells containing the pheromone-inducible transcriptional reporter *P_FUS1_-GFP* were exposed to a 0–10 nM gradient in the microfluidics chamber for 2 hours. The mean GFP fluorescence was calculated using the image analysis software CellProfiler to identify cells and calculate the average fluorescence per cell. The data was determined for each of the 8 regions of the chamber and the fluorescence intensity was normalized to the maximum intensity region (red line). Also shown is the fluorescence profile of the Dextran-3000-TRITC tracer dye (blue line).
(0.64 MB EPS)

Figure S4 Mating projection direction determination from bright-field and fluorescence images. A field of *sst2Δ* cells is shown. On the left is the bright-field image, in the middle is the fluorescence image of ConA-Alexa-488 labeling the growing projection, and on the right is the overlay of the two images. The arrows indicate the direction of the mating projection determined manually from information in the images.
(0.63 MB EPS)

Appendix S1 (0.11 MB DOC)

Table S1 (0.03 MB DOC)
