# Supplementary material for: Robust Spatial Sensing of Mating Pheromone Gradients by Yeast Cells
Source: PLoS One. 2008 Dec 4;3(12):e3865. doi: 10.1371/journal.pone.0003865 (PMC2586657; doi:10.1371/journal.pone.0003865)
Supplement: Appendix S1 — (0.11 MB DOC) [file pone.0003865.s005.doc]

**APPENDIX S1**

**Diffusion profile of alpha-factor labeled with a fluorescent dye.**

We obtained alpha-factor labeled with the fluorescent dye Hylite-488 [1]. In side-by-side experiments, we compared the diffusion profile of the labeled alpha-factor with the tracer dye Dextran-3000-TRITC. The profiles showed good agreement within the experiment-to-experiment margin of error indicating that the diffusion properties of fluorescently-labled alpha-factor were similar to those of the Dextran-3000-TRITC in the microfluidics chamber (Fig. S1).

**Projection directional accuracy in the presence and absence of ConA-Alexa-488.**

We wished to test whether the presence of ConA-Alexa-488 might affect the ability of yeast cells to sense and project up the alpha-factor gradient. We performed experiments in the 0 – 100 nM gradient in the absence of ConA-Alexa-488 and compared the results with the experiments using ConA-Alexa-488 (from Figure 2B). In Figure S2, we measured the proportion of cells that were aligned versus unaligned with the gradient, and the resulting numbers were statistically similar in the two sets of experiments.

**Estimate of alpha-factor concentration in the microfluidics chamber using a *P_FUS1_-GFP* transcriptional reporter strain.**

One concern was that alpha-factor could be sticking to the tubing and chamber so that the actual pheromone concentrations in the chamber were lower than the estimated concentrations. We compared the dose-response of a strain containing an integrated *P_FUS1_-GFP* transcriptional reporter in a microfluidics chamber versus in liquid culture. In the Figure S3, we show a typical dose-response curve from a 0 – 10 nM gradient generated in the microfluidics chamber. The responding cells produced GFP, which was quantitated by image analysis [2]. We determined the average GFP fluorescence per cell in each of the 8 regions of the chamber. From this experiment, we estimated a half-maximal dose, K_0.5_ ~ 4 nM, in the microfluidics chamber. By comparison, when the dose-response experiments were repeated in liquid culture, we obtained the value K_0.5_ = 3 nM, which is consistent with past experiments [3]. This good agreement suggests that little if any alpha-factor was lost in the microfluidics experiments. We believe that the relatively fast flow-rate, the use of rich YPAD media, and pre-running the alpha-factor containing media in the tubing all helped to minimize the loss of alpha-factor.

**Determining projection direction using bright-field and fluorescence images.**

We used information from both bright-field images of the cell morphology and fluorescence images of ConA-Alexa-488 labeling newly synthesized cell wall glycoproteins to determine the direction of the mating projection. For some of the mutant cells, the direction of the projection was challenging to determine. In Figure S4, we show an example of a field of *sst2Δ* cells and how the morphological shape (i.e. tip of mating projection) and the location of the fluorescence (i.e. direction of center of fluorescence) were used to determine the projection direction with assistance from the image overlay.

# Description of generic model of yeast cell polarization.

The mathematical model centers on the spatial dynamics of the heterotrimeric and Cdc42 G protein cycles. The first four equations of the model represent the dynamics of the heterotrimeric G protein cycle, and the last two equations represent the dynamics of the Cdc42 G protein cycle. The input is the ligand α-factor (L) and the output is active Cdc42 (C42a). This model is based on previous work [4].

We modeled the response of an **a**-cell to α-factor. In the heterotrimeric G-protein cycle, the ligand α-factor (L) binds α-factor receptor (R) to form the active receptor complex (RL). The RL species catalyzes the activation of the heterotrimeric G-protein (G) to form active α-subunit (Ga) and free Gβγ (Gbg). Ga is deactivated to form inactive α-subunit (Gd), which binds to Gβγ to reform the heterotrimer.

In the Cdc42 cycle, the level of Gβγ affects the rate of activation of Cdc42 (C42) to its active form (C42a) through the cooperative Hill term ($\frac{k_{0}}{1+(\beta[\text{Gbg*}])^{-q}}$, ) which represents the dynamics of Cdc24, an activator of Cdc42 that binds Gβγ but is not explicitly represented. Note that *q* is the Hill coefficient and that $1/\beta$ is the Hill half-maximal constant; the negative exponent arises from dividing the numerator and denominator of the standard Hill expression by $\beta[\text{Gbg}*]^{q}$. In addition, there is a positive feedback term in which C42a stimulates it own activation, $\frac{k_{1}}{1+(\gamma[\text{C42a}])^{-h}}$. This term describes the positive feedback loop involving Cdc42, Cdc24, and the scaffold protein Bem1 [5]. The negative feedback loop is implemented through the action of the Cdc42-activated kinase Cla4 which is known to phosphorylate and down-regulate the Cdc42 activator Cdc24 [6]. Equation S6 represents an integral control equation [7] which sets the active level to a steady-state value for an activated response. Finally, we normalize active Cdc42 levels to this steady-state value.

Our model incorporated lateral surface diffusion on the membrane. All the proteins were assumed to have the same surface diffusion coefficient *D_m_*. We did not represent diffusion in the cytoplasm because the small size of yeast cells would enable fast mixing of cytoplasmic proteins by diffusion. We assumed that the cell has an axi-symmetric shape with the direction for the gradient of L as the axis of symmetry.

# Model:

$\frac{\partial\text{[R]}}{\partial t} =D_{m}\nabla_{s}^{2}[\text{R}]-k_{RL} [\text{L}][\text{R}]+k_{RLm} [\text{RL}]-k_{Rd0} [\text{R}]+[C42a]k_{Rs}$ (S1)

$\frac{\partial\text{[RL]}}{\partial t} =D_{m}\nabla_{s}^{2}[\text{RL}]+k_{RL} [\text{L}][\text{R}]-k_{RLm} [\text{RL}]-k_{Rd1} [\text{RL}]$ (S2)

$\frac{\partial\text{[G]}}{\partial t} =D_{m}\nabla_{s}^{2}[\text{G}]-k_{Ga} [\text{RL}][\text{G}]+k_{G1} [\text{Gd}][\text{Gbg}]$ (S3)

$\frac{\partial\text{[Ga]}}{\partial t} =D_{m}\nabla_{s}^{2}[\text{Ga}]+k_{Ga} [\text{RL}][\text{G}]-k_{Gd} [\text{Ga}]$ (S4)

$\frac{\partial[C42a]}{\partial t}=D_{m}\nabla_{s}[C42a]+\frac{k_{0}}{1+(\beta[Gbg*])^{-q}}+\frac{k_{1}}{1+(\gamma[C42a])^{-h}}-\left(k_{2}+k_{3}[Cla4]\right)[C42a]+IC(z)$ (S5)

$\frac{\partial[\text{Cla4}]}{\partial t}=k_{4}\left(\frac{\int_{s} [\text{C42a}] ds}{\int_{s} 1 ds}-[\text{C42a}]_{ss}\right)[\text{Cla4}]$ (S6)

**Initial conditions:**

We may assume that [C42], [R], and [G] are initially equally distributed along the cell surface with a total concentration $C42_{t},\text{ R}_{t}\text{ }$and $G_{t}$ respectively.

**Rate constants:**

$k_{RL} =2\times10^{-3}\text{ (nM)}^{\text{-1}}\text{ s}^{\text{-1}}=2\times10^{6}\text{ M}^{\text{-1}}\text{s}^{\text{-1}}$; $k_{RLm} =1\times10^{-2}\text{ s}^{\text{-1}}$;

$k_{Rs} =\frac{4\text{ mol s}^{\text{-1}}}{SA}\text{ }$; $k_{Rd0} =4\times10^{-4}\text{ s}^{\text{-1}}$; $k_{Rd1} =4\times10^{-4}\text{ s}^{\text{-1}}$; $k_{G1} =1\text{ (mol)}^{\text{-1}}\text{ s}^{\text{-1}}\times\text{SA}$

$k_{Ga} =1\times10^{-5}\text{ (mol)}^{\text{-1}}\text{ s}^{\text{-1}}\times SA$; $k_{Gd} =0.1\text{ s}^{\text{-1}}$;

For the simulations of the *sst2Δ* mutant, $k_{Gd} =0.001\text{ s}^{\text{-1}}$. For the simulations of the *ste2^300Δ^* mutant, $k_{Rd0} =k_{Rd1} =4\times10^{-5}\text{ s}^{\text{-1}}$ and $R_{t}\text{=100,000 mol (molecules)}$.

**Geometry:**

The cell was simulated as an axi-symmetric ellipsoid possessing radii of 2 μm (major axis) and 1 μm (minor axis). The surface area and volume of the cell were $SA=21.5$ μm^2^ and $V=8.4$ μm^3^, respectively.

The input was a linear gradient $L=L_{mid}+L_{slope}(z-z_{0})$ where *z_0_* represents the center of the cell.

**References (Appendix S1)**

1. Toshima JY, Toshima J, Kaksonen M, Martin AC, King DS, et al. (2006) Spatial dynamics of receptor-mediated endocytic trafficking in budding yeast revealed by using fluorescent α-factor derivatives. Proc Natl Acad Sci U S A 103: 5793 - 5798.

2. Carpenter A, Jones T, Lamprecht M, Clarke C, Kang I, et al. (2006) CellProfiler: Image analysis software for identifying and quantifying cell phenotypes. Genome Biology 7: R100.

3. Yi TM, Kitano H, Simon MI (2003) A quantitative characterization of the yeast heterotrimeric G protein cycle. Proc Natl Acad Sci U S A 100: 10764-10769.

4. Yi T-M, Chen S, Chou C-S, Nie Q (2007) Modeling yeast cell polarization induced by pheromone gradients. J Stat Phys 128: 193 - 207.

5. Butty AC, Perrinjaquet N, Petit A, Jaquenoud M, Segall JE, et al. (2002) A positive feedback loop stabilizes the guanine-nucleotide exchange factor Cdc24 at sites of polarization. Embo J 21: 1565-1576.

6. Gulli M-P, Jaquenoud M, Shimada Y, Niederhauser G, Wiget P, et al. (2000) Phosphorylation of the Cdc42 exchange factor Cdc24 by the PAK-like kinase Cla4 may regulate polarized growth in yeast. Mol Cell 6: 1155 - 1167.

7. Iglesias PA, Levchenko A (2002) Modeling the cell's guidance system. Science's STKE 9: RE12.
