# Supplementary material for: Robust Spatial Sensing of Mating Pheromone Gradients by Yeast Cells
Source: PLoS One. 2008 Dec 4;3(12):e3865. doi: 10.1371/journal.pone.0003865 (PMC2586657; doi:10.1371/journal.pone.0003865)
Supplement: Table S1 — (0.03 MB DOC) [file pone.0003865.s006.doc]

## Table S1. Yeast Strains

| **Strain** | Genotype | **Source** |
| --- | --- | --- |
| BY4741 | *MAT****a****, his3Δ1, leu2Δ0, met15Δ0, ura3Δ0* | Open Biosystems |
| TIM007 | Same as RJD863 with *mfα1Δ::URA3, bar1Δ::HYG* | This Study |
| TIM009 | Same as TIM007 with *ste2^300Δ^::KAN* | This Study |
| RJD863 | *MAT****a***, *can1-100*, *leu2-,3-112*, *his3-11*,*-15*, *trp1-1*, *ura3-1*, *ade2-1*, *bar1*::*LEU2* | Ray Deshaies (Caltech) |
| TIM012 | Same as RJD863 with *mfα1Δ::URA3, sst2Δ::KAN* | This Study |
| HTY055 | Same as RJD863 with *ste2*::*[STE2-GFP*, *HIS5]* | Hiromasa Tanaka (UC Irvine) |
| HTY069 | Same as RJD863 with *spa2*::*[SPA2-GFP, HIS5]* | Hiromasa Tanaka (UC Irvine) |
| HTY072 | Same as RJD863 with *ste18*::*[STE18-GFP, HIS5]* | Hiromasa Tanaka (UC Irvine) |
| HTY073 | Same as RJD863 with *ste20*::*[STE20-GFP*, *HIS5]* | Hiromasa Tanaka (UC Irvine) |
